# The Effect of Ru on the Evolution of the γ′ Phase in Ni-Al-Ru Alloys

**DOI:** 10.3390/ma15093344

**Published:** 2022-05-06

**Authors:** Shaoyang Wang, Fanqiang Meng, Lu Wang, Hongying Yu, Dongbai Sun

**Affiliations:** 1 School of Materials, Sun Yat-sen University, Southern Marine Science and Engineering Guangdong Laboratory (Zhuhai), Guangzhou 510275, China; wanglu68@mail.sysu.edu.cn; 2 Sino-French Institute of Nuclear Engineering and Technology, Sun Yat-sen University, Zhuhai 519082, China

**Keywords:** ruthenium, microstructure evolution, partitioning behavior, lattice misfit

## Abstract

With the development and wide application of nickel-based single-crystal superalloys, the effect of Ru on the microstructure stability and high-temperature properties of superalloys is becoming increasingly important. In this study, the effect of Ru on the evolution of the γ′ phase in Ni-Al-Ru ternary alloys during aging treatment was analyzed, using a scanning electron microscope and transmission electron microscope, combined with energy-dispersive spectroscopy. The relationship between chemical partition behavior and γ/γ′ lattice misfit was investigated in detail. During the aging process, Ru addition suppressed the growth rate and rafting process of γ′ precipitates, while the effect of Ru on hindering γ′ phase growth was reduced when the Ru content was over 3 at%. Ru preferentially partitioned to the γ phase, and its partitioning ratio to the γ phase increased with a variation in Ru content from 1 at% to 3 at% and decreased for the NiAl6Ru alloy. Additionally, the lattice misfit of all alloys was positive and reduced with the increase in Ru content, which hindered the Ru atoms to diffuse into the γ phase and promoted the shape of γ′ precipitates to change from cubic to spherical.

## 1. Introduction

Nickel-based single-crystal superalloy has been extensively used in turbine blades due to its outstanding ability to retain strength and withstand creep, as well as oxidation at high temperatures. These superior high-temperature properties are mainly attributed to its complex composition, with 10 or more alloying elements, e.g., Al, Ti, Ta, Re, W, to improve the strength and creep resistance of γ′ hard precipitates and γ matrix phase. However, a significant amount of refractory elements often lead to a decrease in the high-temperature stability of the microstructure due to the formation of topological close-packed (TCP) phases, which results in the deterioration of the mechanical performance of superalloys [1]. Therefore, with the development of superalloys, the element of ruthenium (Ru) has been added to enhance the microstructural stability and further improve the strength and creep resistance at high temperatures [2,3]. Reports [4,5,6] have shown that the Ru addition will increase the liquidus temperature of nickel-based single-crystal superalloy and affect its microstructural stability and creep properties. However, the effects of Ru on the γ/γ′ lattice misfit, elemental partitioning behavior between phases, high-temperature microstructural stability, and creep properties are still debatable [7]. It is also well known that the sign and magnitude of lattice mismatch influence the precipitation and rafting behavior of the γ′ phase during high-temperature creep [8,9,10]. It will eventually affect the high-temperature mechanical properties of nickel-based single crystal superalloys.

Regarding the effects of Ru addition on the microstructural evolution, Song et al. [7,11,12] report that the growth and coarsening rate of the γ′ precipitates are increased, and stable rafting microstructures forms during the long-term operation with the addition of Ru. While Guan et al. [13,14] report that the increase in Ru content decreases the size and volume fraction of the γ′ phase, and the shape of the γ′ phase becomes more regular. Moreover, the addition of Ru can also hinder the formation of the TCP phase to improve microstructural stability and creep resistance. Some studies [15,16] show that the effect of Ru on hindering the precipitation and growth of TCP phases through the “reverse partitioning” behavior of refractory elements, but other studies [17,18] indicate that the diffusion of refractory elements depends on their composition gradient. Moreover, the addition of Ru can introduce internal strains and lower the stacking fault energy in the Ni lattice to improve high-temperature creep, but Ru is not used as a solid solution strengthening element in alloy design [19,20,21]. In summary, clarifying the relationships among Ru content, elemental partitioning behavior, and microstructural evolution in superalloys is of vital importance for maximizing the potential effects of this alloying element.

In this study, a series of ternary alloys Ni-Al-Ru with γ and γ′ phases were prepared. The temporal evolution of the microstructure of the alloys with different amounts of Ru addition was investigated experimentally using SEM and TEM. As the influence of other elements was excluded, the response of γ′ morphology and its precipitation behavior showed different rules from studies containing other refractory elements, such as Co, Mo, Re, and W in the alloys. The partitioning behavior of the elements and the γ/γ′ lattice misfit are also discussed in detail. This study provides new insights for further understanding of the role of Ru and a theoretical basis for the design of microstructure and properties of nickel-based single crystal superalloy.

## 2. Materials and Methods

Three experimental alloys, designated NiAl1Ru, NiAl3Ru, and NiAl6Ru, were made by induction melting with high-purity alloying elements (99.95 wt.% Ni, 99.99 wt.% Al, and 99.95 wt.% Ru), under an argon atmosphere, after which the casting was chilled into Φ 30 mm × 10 mm ingots. The composition of the alloys is shown in Table 1. To ensure chemical homogeneity, cast ingots were homogenized at 1300 °C under argon atmosphere for 24 h and then cooled in air. The samples with dimensions of 10 mm × 10 mm × 2 mm were cut from the ingots by wire electrical discharge machining. Then, an aging procedure was carried out at 900 °C under an argon atmosphere for 1 h, 4 h, 24 h, and 129 h, respectively, followed by water quenching to room temperature. Aged samples were grounded and polished and then electrolytically etched using a mixed solution of 34 mL H_2_SO_4_, 42 mL H_3_PO_4_, and 24 mL H_2_O; the voltage was 4 V, with the etching time of 3 s. The morphological evolution of the samples was studied employing a scanning electron microscope (SEM), and γ′ precipitates were measured with ImageJ software based on at least 1000 particles. The finer structure of the alloys after heat treatment was analyzed with a transmission electron microscope (TEM) on thin foils. The foils were grounded manually and prepared by a precision ion polishing system (PIPS II 695) at 3.0–5.5 keV, with beam angles down to 2°. Microstructure analysis was conducted using a JEM-2100 TEM operating at 200 kV. In order to understand the partitioning behavior of alloying elements, the compositions of phases were examined by means of energy-dispersive spectroscopy (EDS) equipped in TEM, with an energy resolution of 129 eV and spatial resolution of 10 nm, based on 5 detected positions of γ′ and γ phases, respectively, in each sample. The characteristic X-ray lines of Ni-Kα, Al-Kα, and Ru-Lα were used to record the corresponding EDS elemental map of Al, Ni, and Ru, respectively. Additionally, the lattice misfit between γ and γ′ phases was also analyzed.

## 3. Results and Discussion

### 3.1. Microstructural Characteristics

**Figure 1:**
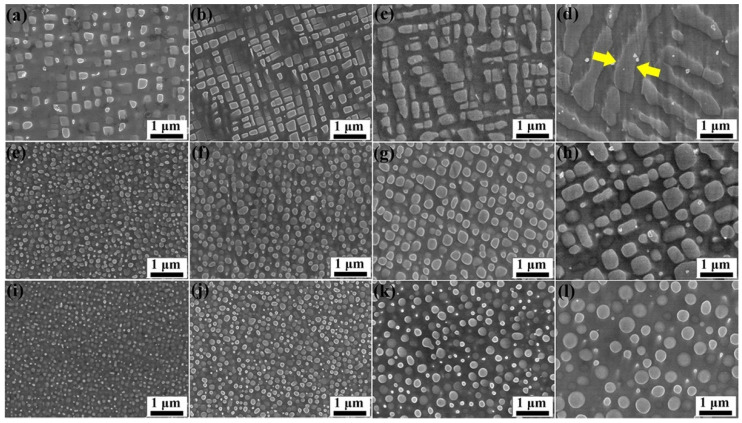
SEM images of NiAl1Ru (**a**–**d**), NiAl3Ru (**e**–**h**), and NiAl6Ru (**i**–**l**) alloys after aging at 900 °C for 1 h (**a**,**e**,**i**), 4 h (**b**,**f**,**j**), 24 h (**c**,**g**,**k**), and 129 h (**d**,**h**,**l**).

The microstructure of the alloys after heat treatment, as shown in Figure 1, mainly consisted of the γ phase and γ′ precipitates. For the NiAl1Ru alloy, cubic γ′ precipitates were homogeneously distributed when aging for a short time, as shown in Figure 1a. With increasing aging time, the size of γ′ grew, and the edges became more parallel to each other after aging 4 h. With prolonged aging time, γ′ precipitates grew rapidly and arranged in lines in perpendicular directions, and the distance between the adjacent γ′ precipitates in the arrangement direction decreased gradually (Figure 1c); finally, γ′ precipitates linked with each other and grew into strip shape after aging 129 h, that is, γ′ precipitates began rafting (Figure 1d). For the alloys with higher Ru content, γ′ precipitates were spherical when the aging time was short (Figure 1e,i), and as the aging process advanced, γ′ precipitates became coarser; therefore, two evolutionary directions emerged for γ′ precipitates. For the NiAl3Ru alloy, γ′ precipitates gradually transformed from spherical into cuboidal shapes (Figure 1g,h). After aging for129 h, the sizes of γ′ precipitates were distributed unevenly, with small particles surrounding the large ones, and the amount of the precipitates reduced; thus, it can be inferred that the growth of γ′ precipitates obeys the Ostwald ripening, as shown in Figure 1h [22]. However, for the alloy with 6 at% Ru, γ′ precipitates showed spherical shapes during the whole aging process in this study (Figure 1i–l). It is clear that the addition of Ru hinders the rafting process of γ′ precipitates during the aging process.

The mean size of γ′ precipitates was analyzed using ImageJ software, and the results are shown in Table 2. Here, in order to obtain the accurate γ′ precipitate size, only the width of the rafted γ′ precipitate was measured, as marked between the two yellow arrows in Figure 1d. From the data, it can be found that the γ′ size increased with the prolonging of aging time but decreased with the increase in Ru content. Based on the fact that the growth of γ′ precipitates is controlled by elemental diffusion [23], the growth of γ′ precipitates can be described with Lifshitz–Slyosov–Wagner (LSW) theory according to Equation (1) [24].

(1) $${\overset{-}{r}}^{3}-{{\overset{-}{r}}_{0}}^{3}=\mathrm{kt}$$

where ${\overset{-}{r}}_{0}$ and $\overset{-}{r}$ are the initial and instantaneous particle radius, respectively, and *k* and *t* are the growth rate and time, respectively.

**Figure 2:**
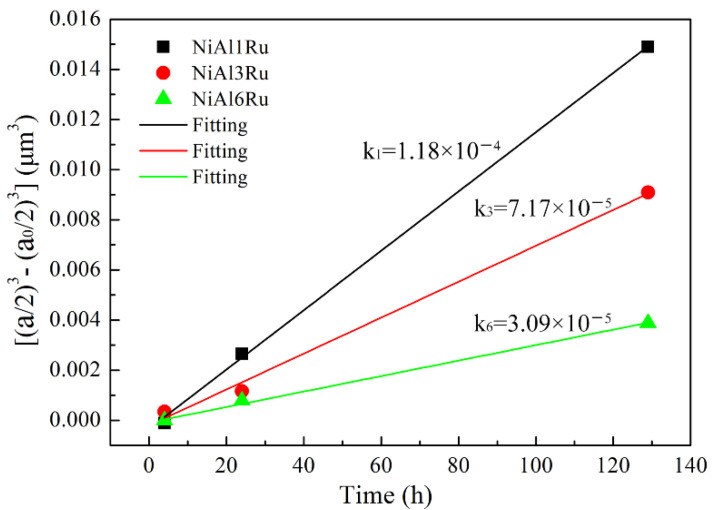
Relationship between mean size of γ′ precipitates and aging time.

For cuboidal particles, $\overset{-}{r}$ is replaced by *a*/2, and ${\overset{-}{r}}_{0}$ is replaced by *a*_0_/2. Additionally, in this study, *a* represents the average side length of the cubic γ′ precipitates after aging treatment, and *a*_0_ is the average side length of the cubic γ′ precipitates after solution treatment, and in this paper, we used the particle size after aging for 1 h as *a*_0_. Therefore, the particle size in Table 2 is presented as a plot of (*a*/2)^3^–(*a*_0_/2)^3^ versus *t*, as shown in Figure 2. The plots show excellent linear fittings of growth rates *k* of the γ′ precipitates for the NiAl1Ru, NiAl3Ru, and NiAl6Ru alloys aged at 900 °C. The values of *k* were calculated and marked in Figure 2, illustrating the diffusion coefficient. Figure 2 shows that the slopes varied with the Ru content, and a lower Ru content corresponded to a larger slope—namely, a faster rate of coarsening in γ′ precipitates. Since the only difference between the alloys was the Ru content, it can also be inferred that the addition of Ru hinders the growth of γ′ precipitates.

### 3.2. The Partitioning Behavior of Ru

**Figure 3:**
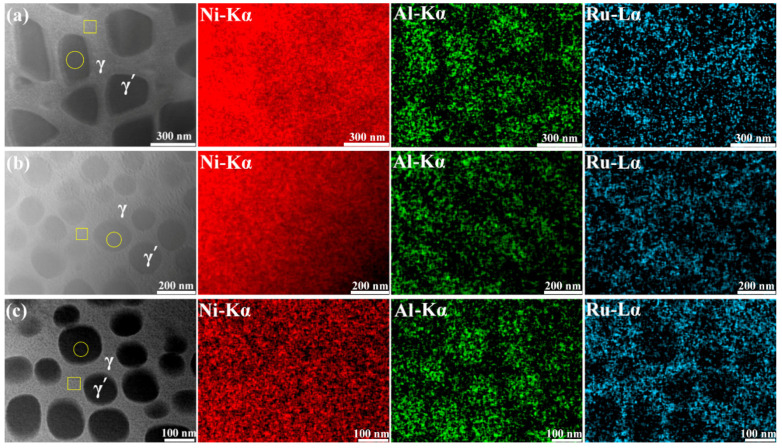
HAADF STEM images and EDS mappings of elements distribution for samples NiAl1Ru (**a**), NiAl3Ru (**b**), and NiAl6Ru (**c**) after aging for 4 h (yellow circles and boxes represent the EDS detecting location on γ′ and γ phases, respectively).

**Figure 4:**
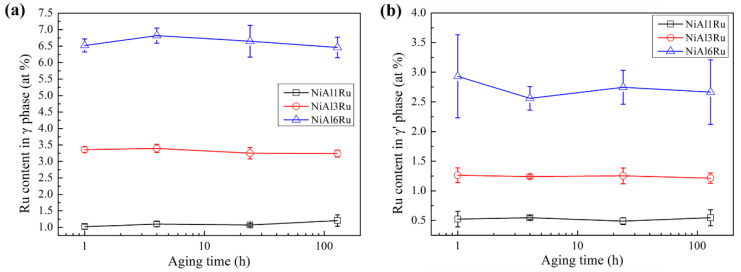
The content of Ru in γ phase (**a**) and γ′ phase (**b**) of the alloys after heat treatment. The horizontal x-axis is converted to logarithmic coordinates for clarity.

The microstructure of the samples was observed with TEM, and the distributions of elements in the γ and γ′ phases were analyzed using EDS in TEM; Figure 3 shows the HAADF STEM images of microstructures and the corresponding EDS mappings of elements distribution in samples NiAl1Ru, NiAl3Ru, and NiAl6Ru after aging for 4 h. It can be seen that the difference in Ni distribution in the two phases was not obvious, and Al mainly existed in the γ′ phase. Ru partitioned preferentially to the γ phase, and its content in the two phases was measured, as shown in Figure 4. The Ru concentrations in both γ and γ′ phases increased with the increase in Ru addition, while the aging process had a slight influence on the distribution of Ru. However, for the NiAl6Ru alloy, the value of Ru content in γ′ fluctuated greatly with aging time, which mainly results from the small particle size, causing low measurement accuracy.

**Figure 5:**
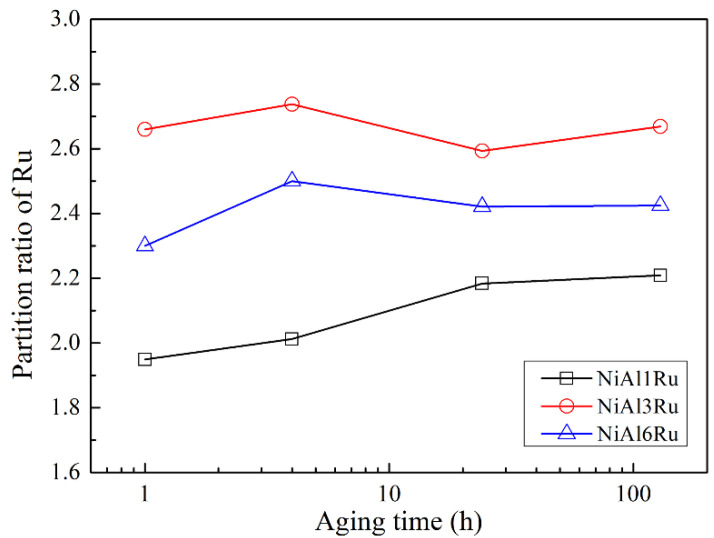
The partition ratios of Ru between γ and γ′ phases of the alloys after heat treatment. The horizontal x-axis is converted to logarithmic coordinates for clarity.

In order to analyze the partitioning behavior of Ru between γ and γ′ phases, the partition ratios of Ru in the two phases are defined as $f_{i}=\frac{x_{i}^{\gamma }}{x_{i}^{\gamma \prime }}$, where *i* represents each of the constituent elements. Since $x_{i}^{\gamma }$ and $x_{i}^{\gamma \prime }$ represent the mole concentration of element *i* in γ and γ′ phases, respectively, partition ratios greater or less than unity indicates preferential partitioning to either the γ or γ′ phase [25]. The results in Figure 5 show that, with the increase in Ru addition, the partition ratio of Ru attributed to γ increased, reaching the maximum value for the alloy with 3 at% Ru in this study. By contrast, the aging treatment slightly affected the partition of Ru, with the partition ratio slightly increasing as the aging time advanced, due to the growth of the γ′ phase with more Ru elements expelled to the γ phase. In addition, with the increase in Ru content to over 3 at%, the ratio of Ru existing in the γ′ phase became higher, so it can be inferred that the addition of more Ru (>3 at%) to the alloys reduces its effect on hindering γ′ phase growth.

**Figure 6:**
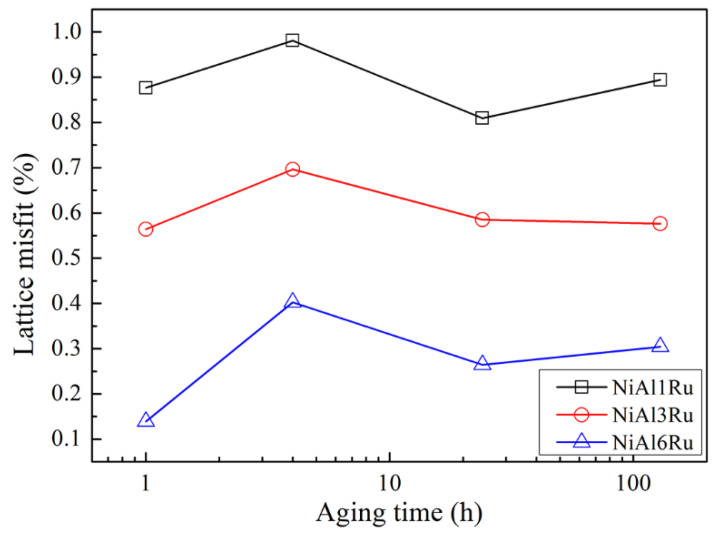
Lattice misfit between γ and γ′ phases after aging at 900 °C over different times. The horizontal x-axis is converted to logarithmic coordinates for clarity.

It is known that the addition of Ru and its partitioning behavior between γ and γ′ phases are crucial to the γ/γ′ lattice misfit, microstructure evolution, and high-temperature mechanical properties [7]. In this study, with the increase in Ru addition, the shapes of γ′ precipitates transformed from cubic into spherical, but this is contrary to the results of other studies in the literature [12,13], indicating that, with the increase in Ru content, the morphology of γ′ precipitates evolves from spherical to intermediately shape and to cuboidal, due to the more negative lattice misfit introduced by the increase in Ru content. Here, the lattice misfit δ between γ and γ′ phases is calculated according to Equation (2) [9].

(2) $$\delta =\frac{{2(a}_{\gamma \prime }-a_{\gamma })}{{(a}_{\gamma \prime }{+a}_{\gamma })}$$

where $a_{\gamma }$ and $a_{\gamma \prime }$ are the lattice constant of γ and γ′ phases, respectively. Additionally, according to the phase composition the lattice constant of the γ and γ′ phases can be estimated by the following Equations (3) and (4) [12].

(3) $$a_{\gamma }=3.524+0.0196C_{C_{o}}+0.110C_{C_{r}}+0.478C_{M_{o}}+0.444C_{W}+0.441C_{R_{e}}+0.3125C_{R_{u}}+\;0.179C_{A_{l}}+0.422C_{T_{i}}+0.7C_{T_{a}}+0.7C_{N_{b}}$$

(4) $$a_{\gamma^{\prime }}=3.57-0.004{C^{\prime }}_{C_{r}}+0.208{C^{\prime }}_{M_{o}}+0.194{C^{\prime }}_{W}+0.262{C^{\prime }}_{R_{e}}+0.1335{C^{\prime }}_{R_{u}}+0.258{C^{\prime }}_{T_{i}}+\;0.5{C^{\prime }}_{T_{a}}+0.46{C^{\prime }}_{N_{b}}$$

where $C_{i}$ represents the atomic fraction of *i* element. In this study, the alloys only consisted of Ni, Al, and Ru, so it can be inferred that for the basic alloy without Ru, the lattice misfit was positive due to the larger atomic radius of Al in the γ′ phase. It is clear that, with the addition of Ru, the lattice parameter of the γ phase grew faster than that of the γ′ phase, due to the preference of Ru partitioning to the γ phase and a larger coefficient multiplying the Ru concentration for the γ phase. Additionally, a previous study [26] found that, in Ni-Al-Ru alloys, Ru resides on the Ni site in the γ phase, resulting in an outward relaxation of Ni lattice and on the Al site in the γ′ phase, which, in turn, leads to an inward relaxation of Ni lattice. Hence, with the increase in Ru content, the lattice misfit became smaller, as shown in Figure 6, and for all of the alloys, the misfit was positive. Additionally, with the increase in aging time, the misfit changed slightly. Therefore, for the alloys with low Ru concentrations, the lattice misfit was large, and the γ′ phase had a cubic shape, tending to raft easily, as shown in Figure 1a–d. For the alloys with 3 at% Ru, the lattice misfit became smaller, and under weaker lattice elastic energy, the γ′ phase was spherical at the beginning of precipitation but grew into a cubic shape with rounded corners during the aging process, as shown in Figure 1e–h. For the alloys with 6 at% Ru, the lattice misfit was the smallest, leading to the smallest lattice elastic energy; thus, the γ′ phase was spherical during the whole aging process, as shown in Figure 1i–l. Meanwhile, the elastic strain introduced by lattice misfit leads to distortion at the interface plane and diversion of γ and γ′ crystal angles. This, in turn, causes the γ′ phase to have a greater tendency toward agglomerating and coarsening at higher temperatures [27]. Then, it can also be inferred that, due to the reduction in the lattice misfit, it becomes difficult for the Ru atoms, which are rejected from the γ′ phase, to diffuse into the γ phase [28]. Therefore, the growth rate of the γ′ phase was reduced with the increase in Ru content. However, it can be inferred that if the aging time is long enough, the γ′ phase in the alloy NiAl6Ru will also grow into cubic under a small amount of lattice elastic energy that is not zero.

## 4. Conclusions

The effect of Ru on the evolution of the γ′ phase in the Ni-Al-Ru ternary alloys was studied in terms of variations in Ru content and aging heat treatment. Additionally, the results are concluded as follows:

1. The addition of Ru can hinder the coarsening and rafting process of γ′ precipitates during aging treatment, but the effect of Ru on hindering γ′ phase growth reduced when the Ru content was over 3 at%;
2. Ru mainly existed in the γ phase, and its partition ratio to the γ phase increased with the variation in Ru content from 1 at% to 3 at% and decreased for the NiAl6Ru alloy;
3. The lattice misfit of the alloys was positive and was reduced with the increase in Ru content, which hindered the Ru atoms to diffuse into the γ phase and promoted the shape of γ′ precipitates to change from cubic to spherical.

## Figures and Tables

**Table 1:** Composition of the designed alloys (at%).

**Alloy**	**Al**	**Ru**	**Ni**
NiAl1Ru	17.82	1.00	Balance
NiAl3Ru	17.46	3.00	Balance
NiAl6Ru	16.92	6.00	Balance

**Table 2:** Mean sizes of γ′ precipitates after aging treatment at 900 °C over different times.

Aging Time (h)	Mean Particle Size (μm)		
	NiAl1Ru	NiAl3Ru	NiAl6Ru
1	0.18 ± 0.053	0.12 ± 0.028	0.11 ± 0.024
4	0.17 ± 0.053	0.16 ± 0.041	0.13 ± 0.035
24	0.30 ± 0.108	0.22 ± 0.056	0.20 ± 0.051
129	0.50 ± 0.331	0.42 ± 0.131	0.32 ± 0.095

## Data Availability

The data presented in this study are available upon request from the corresponding author.
